# Chirurgische Notfälle während der COVID-19-Pandemie

**DOI:** 10.1007/s00104-023-01832-x

**Published:** 2023-03-01

**Authors:** Benjamin Prokein, Michael Dau, Thomas Mittlmeier, Clemens Schafmayer, Bernhard Frerich

**Affiliations:** 1 Klinik für Mund‑, Kiefer- und Plastische Gesichtschirurgie, Universitätsmedizin Rostock, Schillingallee 35, 18055 Rostock, Deutschland; 2 Klinik für Unfall‑, Hand- und Wiederherstellungschirurgie, Universitätsmedizin Rostock, Schillingallee 35, 18055 Rostock, Deutschland; 3 Klinik und Poliklinik für Allgemein‑, Viszeral‑, Thorax‑, Gefäß- und Transplantationschirurgie, Universitätsmedizin Rostock, Schillingallee 35, 18055 Rostock, Deutschland

**Keywords:** Coronavirus, Triage, SARS-CoV-2, Chirurgische Notaufnahme, Personalplanung, Coronavirus, Triage, SARS-CoV-2, Surgical emergency department, Workforce management

## Abstract

**Hintergrund:**

Innerhalb weniger Monate hatte sich COVID-19 weltweit verbreitet. Studien konnten zeigen, dass es in diesem Zusammenhang zu einem Rückgang ärztlicher Konsultationen kam. Vor dem Hintergrund neuer Diskussionen über erneute Einschränkungsmaßnahmen bei steigenden COVID-19-Fallzahlen, soll diese Studie die Auswirkungen der COVID-19-Pandemie auf chirurgische Notfälle der Universitätsmedizin Rostock untersuchen und die Fallzahlen denen der Vorjahre gegenüberstellen.

**Material und Methode:**

Ziel der Studie war es, die Fallzahlen der chirurgischen Notaufnahme der Universitätsmedizin Rostock im Zeitraum 2020 und 2021 denen der letzten beiden Vorjahre (2018, 2019) gegenüberzustellen. Weiterhin erfolgte die Untersuchung des Einflusses der COVID-19-Fallzahlen auf die Fallzahlen der Notaufnahme.

**Ergebnisse:**

Insgesamt konnten die Daten von 74.936 Patientenfällen in die Studie eingeschlossen werden. Es zeigte sich ein hochsignifikanter Rückgang der chirurgischen Notfälle während der COVID-19-Pandemie (*p* < 0,001). Diese zeigten eine hochsignifikante negative Korrelation mit den COVID-19-Fallzahlen (*p* < 0,001). Die Einschränkungen der Bundesregierung hatten direkten Einfluss sowohl auf die COVID-19-Fallzahlen als auch damit verbunden auf die Fallzahlen der Notaufnahme.

**Schlussfolgerung:**

Es zeigte sich ein direkter Zusammenhang der COVID-19-Fallzahlen auf die Fallzahlen der chirurgischen Notaufnahme. Bei insgesamt schwieriger Personalplanung während einer Pandemie kann dies wichtige Hinweise für ein geeignetes Personalmanagement bei ähnlichen zukünftigen Ereignissen liefern.

## Abstract (en)

**Background:**

Within a few months coronavirus disease 2019 (COVID-19) spread around the world. Some studies showed a significant reduction in medical consultations. Due to new discussions about restrictions in the case of rising COVID-19 case numbers, this study investigated the consequences of the COVID-19 pandemic on surgical emergencies at the Rostock University Medical Center and compared the results to the years before.

**Material and methods:**

The aim of the study was to compare the case numbers of the surgical emergency department of the Rostock University Medical Center from the period 2020 and 2021 to the years before (2018, 2019). Furthermore, the consequences of COVID-19 case numbers for the case numbers of the surgical emergency department were investigated.

**Results:**

A total of 74,936 patient cases could be included in this study. The results showed a significant reduction of surgical emergencies during the COVID-19 pandemic (*p* < 0.001). There was a significant negative correlation with the COVID-19 case numbers (*p* < 0.001). The restrictions of the government showed a direct influence on COVID-19 case numbers and emergency visits.

**Conclusion:**

The results showed a correlation of COVID-19 case numbers with the case numbers of the surgical emergency department. Due to difficult workforce planning during a pandemic, this can be important information for workforce management in similar future events.

## Kernaussagen

1. Die Einschränkungsmaßnahmen der Bundesregierung führten zum Rückgang der COVID-19-Fallzahlen und damit verbunden zu einem Rückgang der Fallzahlen in der chirurgischen Notaufnahme.
2. Während der Pandemie (2020, 2021) zeigten sich weniger chirurgische Notfälle als in den beiden Vorjahren (2018, 2019).
3. Weniger Notfälle entlasten das medizinische Personal.
4. Bei schwieriger Personalplanung aufgrund krankheitsbedingten Personalausfalls, kann eine frühzeitige Personalverschiebung in sensible Bereiche (z. B. Intensivstation) geplant werden.
5. Vor dem Hintergrund neuer COVID-19-Wellen oder anderer Pandemien können Einschränkungsmaßnahmen eine gezielte Personalentlastung im Krankenhaus bewirken, wodurch einer Triagierung durch Personal frühzeitig entgegengewirkt werden kann.

## Hintergrund und Fragestellung

Innerhalb weniger Monate nach dem ersten Auftreten des „severe acute respiratory syndrome coronavirus 2“ (SARS-CoV-2) in Wuhan (China) hatte sich das Virus weltweit verbreitet. Die hohen Inzidenzzahlen führten u. a. auch in Deutschland zu einer Einschränkung des öffentlichen Lebens.

Studien aus dem Gebiet der Inneren Medizin zeigen, dass mit Erhöhung der Infektionszahlen ein Rückgang der stationären Aufnahmen von Patienten mit akutem Myokardinfarkt und durchgeführter perkutaner Interventionen auftrat [1]. Eine Reduktion stationär eingelieferter Patienten mit akutem Koronarsyndrom war auch in Norditalien und Österreich zu verzeichnen [2, 4]. In den USA waren Herzkatheterinterventionen bei ST-Hebungsinfarkten rückläufig [3]. Hierbei wird ein möglicher Zusammenhang mit einer reduzierten Inanspruchnahme des Rettungsdienstes aus Angst vor SARS-CoV-2-Infektionen diskutiert [1, 3].

Boserup et al. berichten von einem signifikanten Rückgang an Vorstellungen in der Notaufnahme im Zeitraum März/April 2020 in den USA [5]. Eine weitere amerikanische Studie berichtet von einer Abnahme um 42 % in den Monaten März/April 2020 im Vergleich zum Vorjahr. Der größte Rückgang zeigte sich hierbei bei Patienten ≤ 14 Jahre, weiblichem Geschlecht sowie im Nordosten der USA [6]. Eine Studie aus der Türkei konnte ebenfalls eine Senkung um 25 % feststellen [7]. Ebenso wurde ein Einfluss auf Krebserkrankungen diskutiert [8].

Im Rahmen der Pandemie zeigten sich temporär an der Universitätsmedizin Rostock personelle Engpässe, aufgrund von COVID-19-assoziierten Krankheitsausfällen. Wie auch in anderen Kliniken wurden planbare Operationen verschoben. Anselm et al. konnten zeigen, dass in Rheinland-Pfalz neben den intensivmedizinischen Belegungen auch Normalstationen deutlich mehr belastet waren [9]. Die knappen Personalressourcen stellten weltweit Kliniken vor große Herausforderungen. „Lockdowns“ zur Eindämmung der Infektionszahlen waren in vielen Ländern die Folge. In Spanien konnte dadurch eine Reduktion pädiatrischer Notfälle von 58 % innerhalb von 2 Wochen erzielt werden [10]. Auch in anderen Ländern konnten durch Lockdowns die Fallzahlen in der Notaufnahme reduziert werden [11–14]. Trotz erhöhter psychischer Belastung der Bevölkerung zeigte sich dieses Phänomen auch in psychiatrischen Notaufnahmen [15].

Mit dieser Studie soll untersucht werden, wie sich die Pandemie auf die Fallzahl chirurgischer Notfälle an der Universitätsmedizin Rostock ausgewirkt hat.

## Studiendesign und Untersuchungsmethoden

Mithilfe des klinischen Informationssystems (SAP ISHmed) wurden alle Fälle der chirurgischen Notaufnahme im Zeitraum 01/2018 bis 12/2021 erfasst und in eine Tabelle übertragen. Hierbei erfolgte die Erfassung demographischer Daten sowie die Art der Behandlung (ambulant, teilstationär, stationär). Es wurde untersucht, ob die COVID-19-Fallzahlen des Robert Koch-Instituts (RKI) Einfluss auf die chirurgische Notfallversorgung hatten.

Hierbei wurden die Datensätze von 2018 bis 2019 denen von 2020 bis 2021 in Kalenderwochen (je zwei) gegenübergestellt. Es wurden absolute und relative Häufigkeiten berechnet. Weiterhin erfolgte die Erfassung der behandelnden Fachrichtung. Der Vergleich der Datensätze 2020 und 2021 zu den Vorjahren erfolgte nach Überprüfung auf Normalverteilung durch Erhebung des T‑Tests; *p*-Werte ≤ 0,05 wurden als signifikant angesehen. Eine Korrelation nach Pearson erfolgte, um die COVID-19-Fallzahlen mit Fallzahlen der chirurgischen Notaufnahme zu vergleichen. Die COVID-19-Fallzahlen wurden aus der Datenbank des RKI entnommen [16].

## Ergebnisse

Insgesamt konnten die Daten von 74.936 Patienten in die Analyse einbezogen werden. Ein Fall war hierbei unvollständig. Das Durchschnittsalter lag zwischen 0–104 Jahren mit einem Mittelwert von 38,93 Jahren. Hierbei wurden 56.053 Fälle ambulant, 18.852 stationär und 31 Fälle teilstationär geführt. Es waren 40.835 (54,5 %) männliche sowie 34.100 (45,5 %) weibliche Patienten beinhaltet. Bezogen auf die Fachabteilungen wurden unfallchirurgische (59,6 %), allgemeinchirurgische (14,7 %), MKG-chirurgische (12,4 %), kinderchirurgische (9,3 %), neurochirurgische (3,9 %) sowie wenige thorax- und kardiochirurgische Notfälle registriert (Tab. 1). Damit waren alle Fachabteilungen der chirurgischen Notaufnahme der Universitätsmedizin Rostock erfasst. In manchen Fällen (z. B. Mittelgesichtsverletzungen, Polytrauma) waren mehrere Fachabteilungen gleichzeitig involviert.

**Table 1:** 

	Absolute Häufigkeit [*n*]	Relative Häufigkeit [%]
**Alter**	(74.936)	–
Min	0	–
Max	104	–
**Geschlecht**	(74.935)	–
Männlich	40.835	54,5
Weiblich	34.100	45,5
**Fallart**	(74.935)	–
Ambulant	56.053	74,8
Stationär	18.851	25,2
Teilstationär	31	0,0
**Fachabteilung**	(81.074)	–
Allgemeinchirurgie	11.914	14,7
Kinderchirurgie	7541	9,3
Neurochirurgie	3152	3,9
Thoraxchirurgie	13	0,0
Unfallchirurgie	48.347	59,6
Kardiochirurgie	35	0,0
MKG-Chirurgie	10.072	12,4

**Figure 1:**
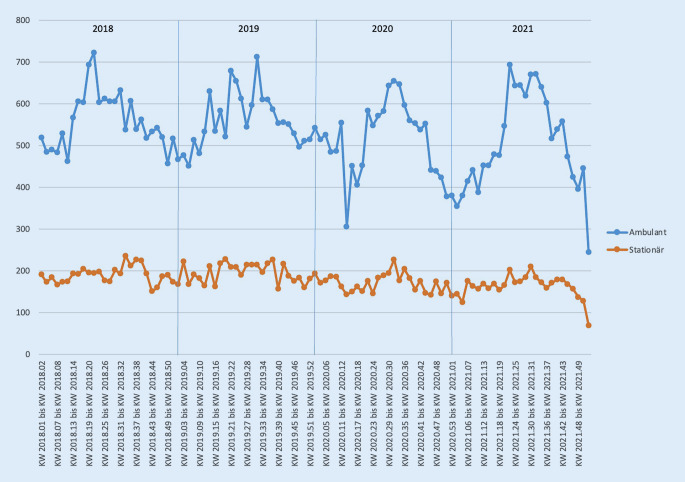

**Figure 2:**
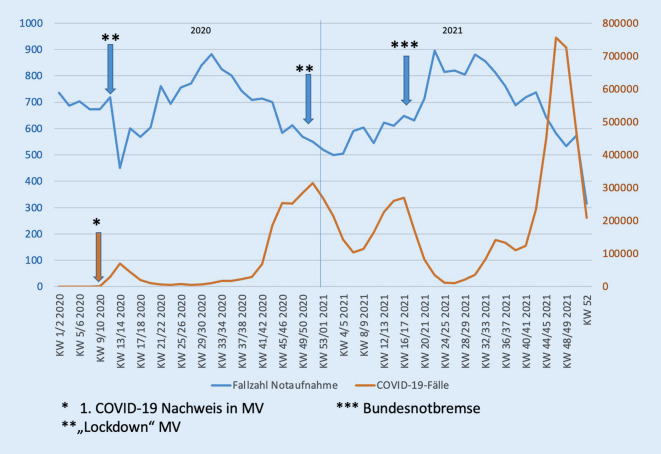

Bei der Korrelation der Fallzahlen der Notaufnahme mit den gemeldeten COVID-19-Fällen des RKI zeigte sich eine hochsignifikant negative Pearson-Korrelation (*p* < 0,001). Beim Vergleich der Fallzahlen von 2020 und 2021 mit denen der beiden Vorjahre (2018, 2019) zeigten sich im T‑Test signifikante Unterschiede. Hierbei lagen keine signifikanten Unterschiede innerhalb der „Prä-COVID-19-Ära“ (2018, 2019) sowie der „COVID-19-Ära“ (2020, 2021) vor. Beim Vergleich der Jahre untereinander zeigten sich jedoch hochsignifikante Unterschiede (*p* < 0,001, Tab. 2). Insgesamt zeigte sich über den Beobachtungszeitraum von 2018 bis 2021 kein signifikanter Rückgang der stationär geführten Patienten. Deutlich wurde der Unterschied jedoch bei den ambulant geführten Notfallpatienten, insbesondere in den Pandemiejahren 2020 und 2021 (Abb. 1). Die Fallzahlen von 2018/2019 lagen hier bei 39.095 und reduzierten sich auf 35.841 in den Jahren 2020/2021. In Abb. 2 kann man den Einfluss der Einschränkungsmaßnahmen auf die COVID-19-Fallzahlen sowie hierbei auch indirekt auf die Fallzahlen der Notaufnahme nachvollziehen.

**Table 2:** 

T‑Test *Fallzahlen*	2018 *19.503*	2019 *19.592*	2020 *17.919*	2021 *17.922*	2018/2019 *39.095*	2020/2021 *35.841*
2018	–	*p* = 0,437	***p*** **=** **0,008**	***p*** **=** **0,004**	–	–
2019	*p* = 0,437	–	***p*** **=** **0,007**	***p*** **=** **0,003**	–	–
2020	***p*** **=** **0,008**	***p*** **=** **0,007**	–	*p* = 0,225	–	–
2021	***p*** **=** **0,004**	***p*** **=** **0,003**	*p* = 0,225	–	–	–
2018/2019	–	–	–	–	–	***p*** **<** **0,001**
2020/2021	–	–	–	–	***p*** **<** **0,001**	–

Die fett hervorgehoben Werte sind hochsignifikant

Die Fallzahlen der einzelnen Jahre sind *kursiv* markiert

## Diskussion

Anhand dieser Daten lässt sich der direkte Einfluss der COVID-19-Fallzahlen auf die Anzahl chirurgischer Notfallpatienten an der Universitätsmedizin Rostock zeigen. Aufgrund der Rolle als Maximalversorger konnte ein Patientenkollektiv von 74.936 einbezogen werden. Es zeigte sich hierbei eine signifikante negative Korrelation der direkten Beziehung der COVID-19-Fallzahlen mit den notfallchirurgischen Fallzahlen. Insgesamt zeigte sich auch ein signifikanter Rückgang der Fallzahlen der Notaufnahme im Zeitraum der Pandemie im Vergleich zu den Vorjahren. Trotz der hohen Fallzahlen ist die Studie durch das monozentrische Design limitiert. Ein größeres Kollektiv mit Kliniken anderer Bundesländer würde die Aussagekraft optimieren.

Wie auch von anderen Autoren beschrieben [1–15], hatte die COVID-19-Pandemie direkten Einfluss auf die Krankenversorgung, was auch chirurgische Notfälle betrifft. Ein Erklärungsmodell könnte die Einschränkung des öffentlichen Lebens durch die Einschränkungsmaßnahmen der Bundesregierung im Rahmen der Pandemie sein. Interessanterweise kam es trotz der einhergehenden erhöhten psychischen Belastung [15, 17–19] auch in psychiatrischen Notaufnahmen zu einem Rückgang der Fallzahlen [15]. Wie in Abb. 2 dargestellt, hatten die Einschränkungsmaßnahmen unmittelbaren Einfluss auf die COVID-19-Fallzahlen und damit verbunden auch auf die Fallzahlen der chirurgischen Notaufnahme. Hierbei wurden u. a. Freizeitaktivitäten wie Sport, Diskobesuche und weiteres reglementiert. Zudem ist durch die Zunahme von Homeoffice das Risiko für Wegeunfälle reduziert worden. Dies könnte ein Erklärungsmodell für den Rückgang an Verletzungen und Traumata darstellen. Weiterhin ist auch eine zurückhaltende Vorstellung in der Notaufnahme durch individuelle Ängste der Patienten (Unsicherheit, mögliche Ansteckung im Krankenhaus oder an öffentlichen Plätzen) zu diskutieren, wie auch andere Autoren vermuten [1, 3].

Am 04.03.2020 meldete das RKI den ersten Fall von COVID-19 in Mecklenburg Vorpommern (MV). Der 1. Lockdown wurde in MV am 17.03.2020 beschlossen. Abb. 2 zeigt hier den direkten Einbruch der Fallzahlen in der chirurgischen Notaufnahme. Ein ähnliches Bild zeigte sich dann im Dezember beim 2. Lockdown.

Dieser Effekt auf die Fallzahlen durch Einschränkungsmaßnahmen zeigte sich auch in anderen Ländern [10–14]. Obwohl die Maßnahmen sich zwischen den Ländern zum Teil unterschieden haben, erzielten alle eine Reduzierung der Fallzahlen. Wärnhjelm et al. konnten allerdings in Helsinki keinen Rückgang chirurgischer pädiatrischer Notfälle verzeichnen, wenn auch die Gesamtzahl an pädiatrischen Notfällen reduziert war [14].

In Rheinland-Pfalz klagten etwa 60 % der Kliniken aufgrund der Personalbelastung über eine eingeschränkte Behandlungskapazität [9]. Durch die reduzierte Inanspruchnahme der chirurgischen Notaufnahme an der Universitätsmedizin Rostock, aufgrund des Rückgangs der Fallzahlen, konnte hier die Belastung des Personals reduziert werden. Vor dem Hintergrund dieser Ergebnisse können zukünftige infektionsbedingte Pandemien rechtzeitig personell strukturiert werden („Ressourcenmanagement“). Eine planbare Verschiebung von Personal in sensible überlastete Bereiche wie z. B. Intensivstationen kann frühzeitig organisiert werden.

Die Bundesnotbremse konnte den o. g. Effekt nicht mehr auslösen. Allerdings zeigte sich bei den Maßnahmen ein deutlicher Rückgang der COVID-19-Fallzahlen durch das RKI.

## Schlussfolgerung

Wie auch von Autoren aus anderen Ländern beschrieben, hatte die COVID-19-Pandemie direkte Auswirkungen auf die Fallzahlen der Notaufnahme [1–15]. Dies betrifft neben internistischen, pädiatrischen und psychiatrischen Patienten auch chirurgische Notfallpatienten. Hierbei besteht eine direkte negative Korrelation zwischen den COVID-19-Fallzahlen und Fallzahlen der chirurgischen Notaufnahme.

Zumindest initial konnte durch die Einschränkungsmaßnahmen die Fallzahl in der chirurgischen Notaufnahme reduziert und damit verbunden eine Entlastung des medizinischen Personals erreicht werden. Auch wenn die Belastung des Personals in der Pandemie groß war und eine Herausforderung darstellte, aufgrund krankheitsbedingter Ausfälle, konnte hier temporär Entlastung erzielt werden. Diese Ergebnisse decken sich auch mit den Erfahrungen aus anderen Ländern [10–14]. Dies ist vor dem Hintergrund kommender Infektionsgeschehen und Pandemien von großer Bedeutung, um hier im Rahmen eines Ressourcenmanagements des medizinischen Personals vorab eine bessere Organisation und Planung durchführen zu können. Weiterhin besteht hierbei auch die Möglichkeit, über Einschränkungsmaßnahmen Fallzahlen in der Notaufnahme reduzieren zu können.
